# Acute myocardial infarction after blunt chest wall trauma with underlying coronary aneurysm: a case report

**DOI:** 10.1186/s12872-018-0861-x

**Published:** 2018-06-18

**Authors:** Xu Guo, Xiaoou Wang, Xinzhong Zhang, Ahmed O. Ahmed, David H. Hsi, Daqing Zhang

**Affiliations:** 1 Department of Cardiology, Shengjing Hospital of China Medical University, #36 Sanhao Street, Heping district, Shenyang City, Liaoning Province 110004 People’s Republic of China; 2 Heart& Vascular Institute, Stamford Hospital, Stamford, CT 06904 USA

**Keywords:** Coronary artery aneurysm, Kawasaki disease, Acute coronary syndrome, Echocardiography

## Abstract

**Background:**

Kawasaki disease is an acute febrile disease with mucocutaneous and cardiovascular involvement affecting infants and young children. Though coronary artery abnormalities are common in Kawasaki disease, no consensus has been reached regarding the treatment of acute coronary artery diseases in this population.

**Case presentation:**

We described a case of myocardial infarction triggered by blunt chest wall trauma in a 20 years old girl. She presented with chest pain and breathlessness with brief syncope, lab results and electrocardiogram findings were consistent with acute myocardial infarction. Chest computer tomography (CT) demonstrated coronary artery calcifications and echocardiography revealed multiple giant left anterior descending aneurysms, suggestive of Kawasaki disease. Subsequent contrast enhanced 3 dimensional coronary computer tomography angiography (CTA) confirmed these findings. We managed this young patient with a conservative strategy. The patient remained symptom free during 2-years follow-ups.

**Conclusions:**

Prompt medical treatment for traumatic myocardial infarction even with underlying giant coronary artery aneurysms can successfully preserve left ventricular function and prevent remodeling with good short term prognosis.

## Background

Kawasaki disease (KD) is an acute febrile disease with mucocutaneous involvement affecting infants and young children first described in Japan in 1967 [1]. Later, KD was found to target coronary arteries and other cardiovascular structures, making the patients vulnerable to coronary artery aneurysms (CAA) and thrombosis [2]. No consensus has been reached regarding the treatment for acute coronary syndrome (ACS) in this population. Here we describe a case of myocardial infarction (MI) triggered by blunt chest wall trauma in a 20-year old patient with possible underlying KD.

## Case presentation

**Fig. 1:**
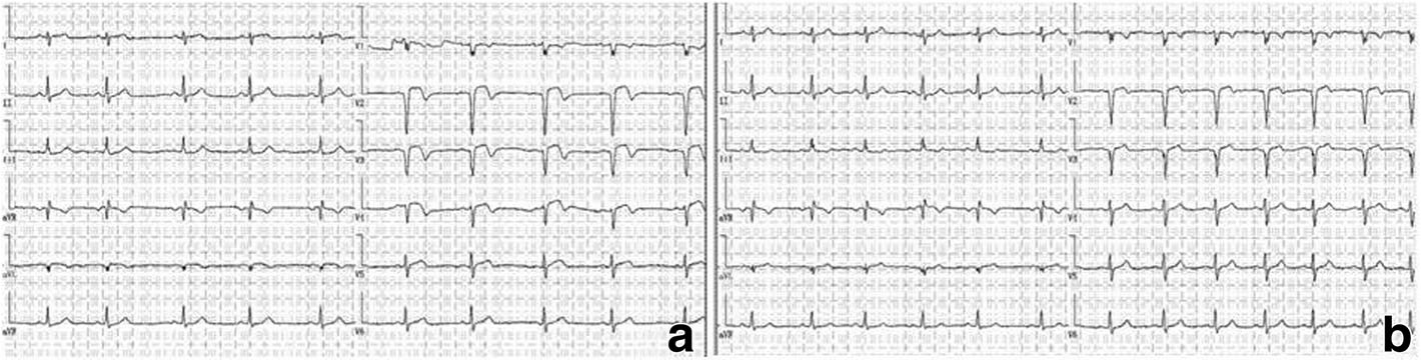
Dynamic ECG changes after blunt chest wall trauma. **a** ECG on admission: sinus rhythm with QS complexes in leads V2 to V3, ST segment elevations and T-wave inversions in leads V2 to V5. **b** ECG at the 2-month follow-up: leads V2 and V3 with QS complex and resolution of the other acute changes

**Fig. 2:**
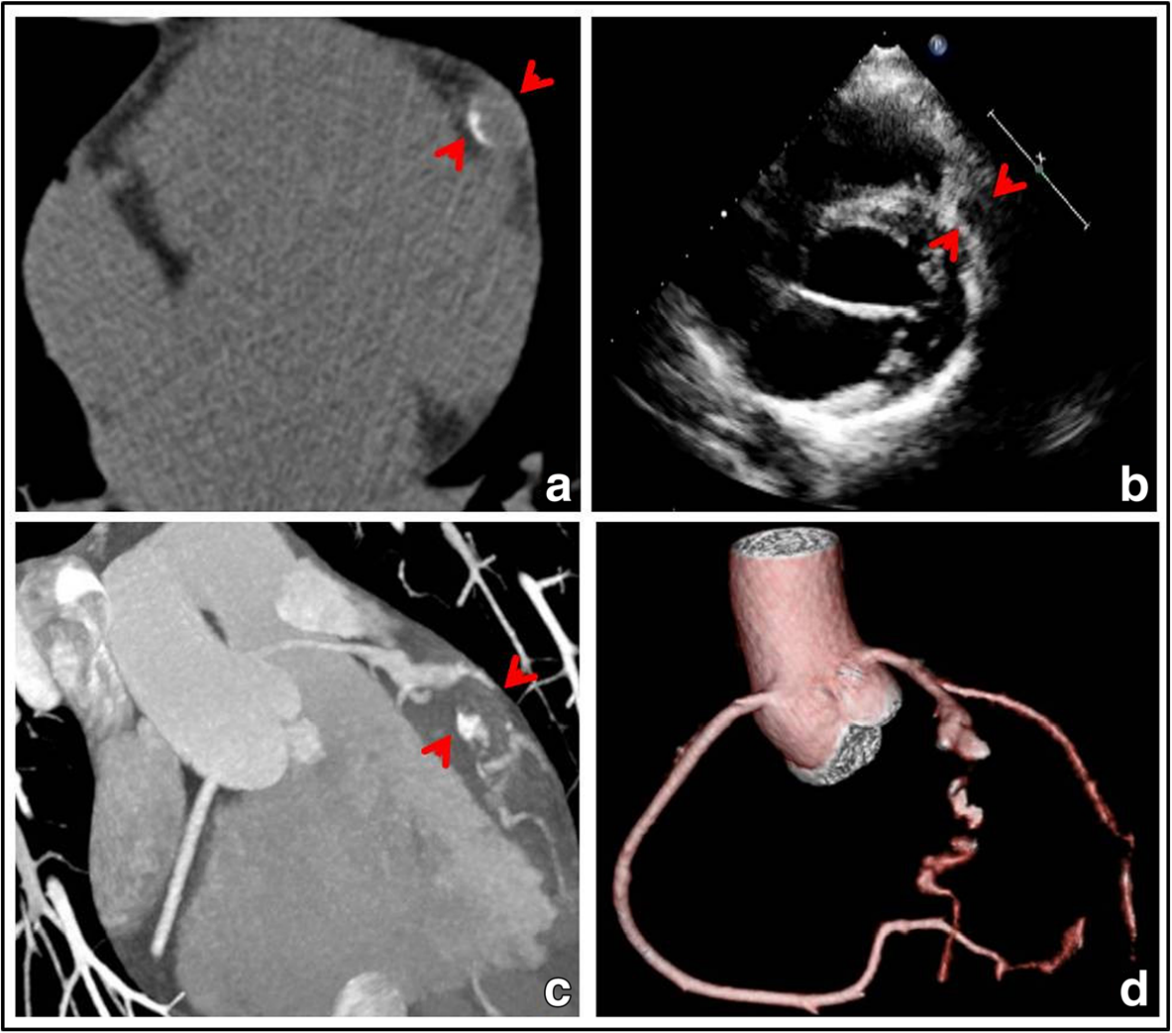
Coronary artery aneurysm of the left anterior descending artery. **a** Chest computer tomography (CT): ringed calcification with 9.8 mm diameter in the left anterior descending (LAD) artery. **b** Trans-thoracic echocardiography: the proximal aneurismal dilation of the LAD artery. **c** Coronary CT angiography(CTA) image in orthogonal plane: LAD artery aneurysm. **d** Coronary CTA with three-dimensional volume rendering: multiple giant aneurysms with lumen occlusion and distal re-canalization in the LAD artery (arrows)

A 20-year-old female patient presented to our hospital with a history of severe and dull substernal chest pain and breathlessness after being hit by a baseball to her chest 2 h ago. Immediately after being hit, she had an episode of syncope briefly without convulsion. The symptoms lasted for half-an-hour and resolved spontaneously. Her vital signs showed blood pressure of 95/64 mmHg and pulse of 70 beats/minute. There was no sign of chest wall penetrating injury. Electrocardiogram (ECG) revealed sinus rhythm with QS complexes in leads V2 to V3, ST segment elevation and T-wave inversion in leads V2 to V5 (Fig. 1a). Laboratory tests showed cardiac Troponin I level of 19.03 ng/ml (normal range < 0.01 ng/ml). Chest computer tomography (CT) revealed no traumatic injury but demonstrated coronary artery calcifications (Fig. 2a). Her parents recalled a history of high fever lasting for several days at the age of 5-year-old. The patient was admitted to our hospital diagnosed of acute traumatic MI. Serum Troponin I was peaked to 20.3 ng/ml, creatine phosphokinase(CK) to 1237 U/L and CK-MB to 101 U/L 12 h after admission. Serum BNP level was normal at 85.9 pg/ml on admission. During hospitalization, serial ECG changes were consistent with an evolving MI. She had frequent ventricular premature beats on the Holter monitor. Trans-thoracic echocardiography revealed normal left ventricular size and mild anterior hypokinesis. Notably, multiple giant left anterior descending (LAD) aneurysms with diameters from 7.5 to 8.5 mm (Fig. 2b) and slow flow velocity were detected. To further assess CAA, we performed contrast enhanced coronary artery computer tomography angiography (CTA)with three-dimensional (3-D)reconstruction of coronary arteries. A ringed calcification in the proximal portion of LAD artery with multiple aneurysms, thrombi and occlusions were visualized (Fig. 2c). CTA also demonstrated multiple aneurysms with beads-on-string appearance in the LAD artery (Fig. 2d). Antinuclear antibody (ANA) was minimally positive (1:10) with unknown significance. Serum levels of C3 (0.864 g/L), C4 (0.912 g/L), CRP (< 1.0 mg/L), ASO (< 25 IU/ml), ESR (15 mm/h), Rheumatoid Factor (< 25 IU/ml) and ANCA were within normal ranges. Those results did not seem to support active vasculitis, rheumatic or immunologic diseases. Based on the typical images and her childhood fever history, we recognized that the patient’s underlying coronary structural abnormality was most likely originated from KD. Considering stable hemodynamic parameters and multiple giant aneurismal dilatations of LAD artery, we managed this young patient with a conservative strategy including dual anti-platelet treatment with aspirin and clopidogrel for at least 1 year, and titrations of captopril and metoprolol to prevent ventricular remodeling. At the 2-month follow-up, the patient remained asymptomatic. ECG showed QS complex only in V2 and V3 leads and other abnormalities were resolved (Fig. 1b). Echocardiography revealed left ventricular diastolic dimension of 48 mm, normal LAD artery velocity, and no ventricular dilatation or akinesis. She remained asymptomatic two year later.

## Discussion and conclusions

According to the third universal definition of myocardial infarction [3], the patient’s present history, dynamic ECG changes, increased serum myocardial biomarkers, and typical coronary artery images were consistent with an acute traumatic MI. Her syncope might have been due to ventricular arrhythmia when the baseball hit her left chest at the vulnerable period of cardiac cycle mimicking commotio cordis. MI after blunt trauma to the chest is a rare clinical condition. The potential mechanisms include coronary artery intimal tearing and hemorrhage, thrombosis and coronary atherosclerotic plaque rupture. The LAD artery has been reported as the most commonly involved coronary artery during blunt trauma. Considering the patient’s stable condition, we performed coronary CTA with 3-D reconstruction. The 3-D coronary CTA images showed this young patient with underlying coronary structural abnormality, which had made her vulnerable for the traumatic MI. Importantly, the large aneurysm calcification in the proximal portion of LAD artery is a typical manifestation of KD [4]. Although coronary angiography, thrombus aspiration and stent placement have been reported to successfully treat patients in critical conditions, serious postoperative complications have also been reported [5, 6]. Giant CAA > 8 mm and large thrombus usually cause underestimation of the diameter or the morphological characteristics, resulting in new aneurysm formation, stent malposition and recurrent thrombosis after stent implantation [7, 8]. In addition, the most commonly used stent in the presence of CAAs is polytetrafluoroethylene(PTFE) membrane covered stent, which has high incidence of restenosis, delayed graft reendothelization, neointimal hyperplasia and side branch occlusion [9]. Thus, the long-term outcome of percutaneous intervention in this population is unpredictable. In young patients, it may be prudent to avoid emergent interventional approach except in life-threatening situations requiring rescue operation. As for coronary artery bypass grafting, Kitamura’s study demonstrated that cardiac event-free rate at 20 and 25 years were only 67 and 60%, respectively [10]. Considering this patient was young and stable, we recommended a conservative strategy with close follow-up. Interestingly, the ECG changes were eventually improved. Echocardiography also showed normal LAD velocity during the 2-month follow-up. We postulated that the patient might have had spontaneous coronary recanalization after the onset of acute MI. Spontaneous recanalization(SR) in AMI might influence the ECG presentation with smaller infarct size and a better prognosis [11, 12]. At this point, the patient is doing well without any post-MI complications.

If the KD in childhood was not treated early with high-dose intravenous immunoglobulin, 1 in 5 children with KD might develop CAA [13]. Although vascular aneurysms associated with KD in childhood may remain clinically silent until adulthood, stenosis and occlusion could progress over years. Angiographic results show that 90% of patients with CAA mostly involved the LAD artery. Risk of MI from KD is peaked in the first 2 years after the initial presentation [14]. However, our patient suffered an acute anterior-septal MI with LAD artery thrombosis at her 20 years of age. We do want to point out the limitation in this patient’s history of febrile illness which could have been due to many other etiologies than KD, in spite of the typical coronary artery aneurysms on the CTA.

It is important to increase the awareness of early identification and appropriate treatment of KD in childhood, which can reduce their future risks of CAAs and MI. Additionally, prompt medical treatment for traumatic MI even with giant CAAs can successfully preserve left ventricular function and prevent remodeling with good prognosis.

## Abbreviations

ACS: Acute coronary syndrome
ANA: Antinuclear antibody
CAA: Coronary artery aneurysms
CK: Creatine phosphokinase
CT: Computer tomography
ECG: Electrocardiogram
KD: Kawasaki disease
LAD: Left anterior descending
MI: Myocardial infarction
